# Spruce sugars and poultry hydrolysate as growth medium in repeated fed-batch fermentation processes for production of yeast biomass

**DOI:** 10.1007/s00449-019-02271-x

**Published:** 2019-12-27

**Authors:** David Lapeña, Pernille M. Olsen, Magnus Ø. Arntzen, Gergely Kosa, Volkmar Passoth, Vincent G. H. Eijsink, Svein J. Horn

**Affiliations:** 1 Faculty of Chemistry, Biotechnology and Food Science, Norwegian University of Life Sciences, P.O. Box 5003, N-1432 Ås, Norway; 2 Department of Molecular Sciences, Swedish University of Agricultural Sciences, P.O. Box 7015, S-75007 Uppsala, Sweden

**Keywords:** Protein hydrolysate, Single-cell protein, Aquaculture, Downstream processing, Repeated batch

## Abstract

The production of microbial protein in the form of yeast grown on lignocellulosic sugars and nitrogen-rich industrial residues is an attractive approach for reducing dependency on animal and plant protein. Growth media composed of enzymatically saccharified sulfite-pulped spruce wood, enzymatic hydrolysates of poultry by-products and urea were used for the production of single-cell protein. Strains of three different yeast species, *Cyberlindnera jadinii*, *Wickerhamomyces anomalus* and *Blastobotrys adeninivorans,* were cultivated aerobically using repeated fed-batch fermentation up to 25 L scale. *Wickerhamomyces anomalus* was the most efficient yeast with yields of 0.6 g of cell dry weight and 0.3 g of protein per gram of glucose, with cell and protein productivities of 3.92 g/L/h and 1.87 g/L/h, respectively. Using the conditions developed here for producing *W. anomalus*, it would take 25 industrial (200 m^3^) continuously operated fermenters to replace 10% of the fish feed protein used in Norway.

**Electronic supplementary material:**

The online version of this article (10.1007/s00449-019-02271-x) contains supplementary material, which is available to authorized users.

## Introduction

A possible future shortage of feed protein will force mankind to explore alternative protein sources that could replace conventional soymeal or fish meal [1]. Several large industrial organic side-streams could potentially be upgraded to feed protein using fermentation processes [2, 3]. Single-cell protein (SCP) refers to cells of microorganisms such as algae, fungi or bacteria which are produced in bioreactors and then used as a protein source in human food or animal feed. Yeast is the most widely accepted microorganism for SCP production [4], because of its superior nutritional quality and acceptability among consumers [5]. Generally, yeast consists of 45–55% (w/w) protein [6], has a beneficial amino-acid profile according to FAO guidelines [7], and is a good source of vitamins [8]. Furthermore, yeast cell walls contain different proportions of mannan-oligosaccharides, ß-glucan, and chitin [9] with potential health-promoting effects [10], such as stimulation of the immune and antioxidant systems in fish [11]. Yeast can be used as whole cell preparations, or the cell wall might be partly broken down to make the protein and the cell wall components more accessible, using mechanical force, hydrolytic enzymes or detergents [7].

A wide variety of substrates have been utilized to cultivate different microorganisms, but to achieve large-scale production and to reduce the cost of SCP, relatively cheap agroindustrial by-products need to be considered as growth medium ingredients [3]. Yeasts can utilize by-products from agriculture, forestry (lignocellulosic residues) and food industries (hydrolysates from meat and fish by-products) as carbon and nitrogen sources for single-cell protein production [12, 13]. Nitrogen might also be sourced from a combination of protein hydrolysates and inorganic nitrogen such as ammonium salts, nitrates and urea, which are relatively cheap nitrogen sources [14]. Yeasts can utilize various inorganic nitrogen compounds as a sole source of nitrogen [15].

In general, fermentation processes can be classified into batch, fed-batch and continuous fermentations. The chosen mode of operation is to a large extent dictated by the kind of products one is aiming for and process economy. A batch fermentation is a closed culture system that contains a finite amount of nutrients that will be consumed after a relatively short period of time, and is thus not ideal for the purpose of SCP production [16]. Fed-batch fermentations are initially established in batch mode and then continuously, or sequentially, fed with fresh medium without removal of culture [17], but have not been established for the production of SCP at a large industrial scale [18]. During continuous fermentations, fresh medium is constantly fed into the fermenters at a constant rate while used media containing microbes are continuously harvested. Therefore, medium conditions do not change over time, and the same growth rate can be maintained throughout the whole cultivation [19]. Continuous fermentation has been the preferred strategy to produce SCP industrially [20, 21]. Another good strategy is called repeated fed-batch fermentation, which is a semi-continuous system of operation where a portion of culture is harvested at regular intervals and replaced by an equal volume of fresh medium [22]. It is considered as one of the best fermentation set-ups for economical SCP production [23]. In contrast to batch fermentation, production of biomass can in this case be prolonged over time, while low dissolved oxygen levels due to the increase of microbial biomass are avoided since cells are withdrawn and replaced with fresh medium. Compared to continuous fermentation, the harvested culture can potentially have a higher concentration of microbial biomass, which can improve the efficiency of the downstream processing.

In this study, media composed of sugars from enzymatically hydrolysed lignocellulosic biomass [24], enzymatically hydrolyzed poultry by-products [13] and urea were used to produce SCP in a semi-continuous mode (repeated fed-batch fermentation). Three different yeast strains belonging to the species *Cyberlindnera jadinii* (anamorph name *Candida utilis*), *Wickerhamomyces anomalus* and *Blastobotrys adeninivorans* (synonym *Arxula adeninivorans*) were tested. Repeated fed-batch fermentations at 1.5 L scale were carried out using benchtop fermenters, where concentrations of cells, substrates, side products and yeast protein were monitored. Production of the best-performing yeast strain, *W. anomalus* J121, was scaled up to 25 L, and the resulting yeast biomass was analyzed for protein and amino-acid content. Finally, the effect of several cell disruptive methods on the yeast morphology was investigated using autolysis, exogenous enzymes and mechanical force.

## Materials and methods

### Materials

Protein-rich enzymatic hydrolysates from chicken and turkey cut-offs were provided by BIOCO AS (Hærland, Norway) and were kept at 4 °C until further use. The poultry hydrolysates contained 50.37 ± 0.03% dry matter out of which 88% was protein, according to product specifications. Glucose was purchased from VWR chemicals (Radnor, United States), and xylose, mannose, lactic acid, acetic acid, ethanol, sulfuric acid, sodium hydroxide, sodium acetate, potassium phosphate, 37% formaldehyde, biotin, glucosamine and Glucanex were purchased from Sigma-Aldrich (Missouri, USA). Urea was kindly provided by Yara International ASA (Oslo, Norway). Kjeltabs for Kjeldahl analysis were purchased from Thomson and Capper Ltd. (Cheshire, UK). Enzymatic hydrolysates of BALI™ pretreated spruce were kindly provided by Borregaard AS (Sarpsborg, Norway). The BALI™ pretreatment [25] involves sulfite pulping of chipped spruce wood (*Picea abies*), with chip size up to 4.5 × 4.5 × 0.8 cm. The carbohydrate composition of the spruce hydrolysate is shown in Supplementary Table S1.

### Microorganisms

*Cyberlindnera jadinii* LYCC 7549*, W. anomalus* J121 (CBS 100,487, Swedish University of Agricultural Sciences, Uppsala, Sweden) and *B. adeninivorans* LS3 (Swedish University of Agricultural Sciences, Uppsala, Sweden) were stored at − 80 °C in cryovials containing 20% (v/v) glycerol and 80% (v/v) YPD medium.

### Shake flask experiments and repeated fed-batch fermentations

#### Shake flask experiments

Shake flask batch fermentations were conducted using media composed of spruce sugar hydrolysate (abbreviated as BALI™, in this study) and different mixtures of poultry hydrolysates and urea. Pre-cultures were prepared by adding 200 µL of a thawed seed culture stored at – 80 °C to 50 mL of the to-be-tested medium in a 250 mL baffled shake flask, followed by incubation at 30 °C, 220 rpm, for 16 h. The initial pH was adjusted to 5.0 using 5 M NaOH or 5 M H_2_SO_4_. Shake flasks containing 50 mL fresh medium were then inoculated with overnight pre-cultures to obtain an initial OD of 0.5, as measured at 595 nm with an UV/VIS spectrophotometer (Hitachi U1900, Tokyo, Japan). The shake flasks were incubated at 30 °C and 220 rpm, and samples were taken at 24 h, for the measurement of pH, cell dry weight (CDW), protein content, and soluble sugars. These experiments were performed in duplicates. All media contained 50 g/L BALI™ glucose and 5.86 g/L nitrogen. The nitrogen was supplied using six different blends of poultry protein hydrolysates and urea. More specifically, 0, 20, 40, 60, 80 or 100% of the nitrogen was supplied by urea.

#### Repeated fed-batch fermentations at 1.5 L and 25 L scale

The bioreactor cultivations were performed in 2.5 L volume glass fermenters (Minifors, Infors, Bottmingen, Switzerland) with working volumes of 1.5 L, and a 42 L Techfors S stainless steel bioreactor (Infors, Bottmingen, Switzerland) with 25 L working volume, both equipped with two six-bladed Rushton impellers. Blends of urea and poultry hydrolysates were autoclaved at 121 °C for 15 min in the bioreactors. BALI™ sugar hydrolysate was autoclaved separately, and aseptically added into the bioreactors.

For repeated fed-batch mode, fresh medium containing poultry hydrolysate and urea was prepared for the 2.5 L bioreactors sterilizing at 121 °C for 15 min. For the 42 L bioreactor, new nitrogen medium was prepared using 80 °C water in 30 L Einar hydrolysis reactors (Belach Bioteknik, Skogås, Stockholm, Sweden) and stored at 4 °C for up to 12–16 h, until use. Also, for the repeated fed-batch experiments, BALI™ hydrolysate was autoclaved separately.

Overnight pre-cultures were prepared by adding 0.2 mL or 1.6 mL of seed culture to 50 mL or 400 mL of the selected medium in 250 mL or 2 L baffled shake flasks for the 2.5 L and 42 L bioreactors, respectively. The pre-cultures were incubated at 30 °C, 220 rpm for approx. 16 h, prior to inoculation of the bioreactors, which were inoculated with 3% (v/v) pre-culture. The temperature for all cultivations was 30 °C. The pH was monitored with a pH probe (Mettler Toledo, Greifensee, Switzerland) and automatically maintained at 5.0 by controlled addition of 5 M NaOH or 5 M H_2_SO_4_. Dissolved oxygen (DO) was set at 30% saturation and regulated by automatic adjustment of the stirrer speed (300–1250 rpm). Cultures were aerated through a sparger at an initial rate of 1.5 L/min or 25 L/min (1 VVM) and a maximum rate of 3 L/min or 50 L/min (2 VVM), for the 2.5 L and 42 L bioreactors, respectively. CO_2_ and O_2_ analysis was performed with a FerMac 368 off-gas analyzer (Electrolab Biotech, Tewkesbury, UK) for the 2.5 L bioreactors and an Infors HT Gas Analyzer (Infors, Bottmingen, Switzerland) for the 42 L bioreactor. Foam was controlled via a foam sensor with two times diluted Glanapon DB 870 antifoam (Busetti, Vienna, Austria). Fermentation data were recorded using IRIS process control software (Infors). The repeated fed-batch fermentation was carried out using a *V*_out_/*V*_*f*_ value of 0.75 (i.e., 75% of the total volume was harvested and replaced by fresh medium in each cycle). The total cultivation time was kept at 72 h or 76 h using cultivation cycles of 8 h (7 cycles) or 12 h (5 cycles), respectively. The first harvest after the initial batch-phase always occurred after 16 h. The cultivation broth was aseptically collected, and a sterilized or pasteurized (the nitrogen medium fraction for the 42 L bioreactor) fresh medium was added into the fermenters with the use of a peristaltic pump connected to the inoculation port. During the fermentation experiments, samples were taken every 4 h for analysis of soluble medium components and yeast biomass.

### Downstream processing

Yeast produced during the repeated fed-bath fermentation in the 42 L bioreactor was collected and kept at 4 °C. The broth, containing the medium and the yeast cells, was centrifuged using a GEA Westfalia Separator Easyscale 10.S (GEA, Bönen, Germany) with a flowrate of 70 L/h and discharge every 120 s. The phase containing the yeast was resuspended in water (1:1, v/v) and washed one time with tap water using a flowrate of 50 L/h and 90 s discharge time. Different aliquots were stored as a yeast paste (i.e., a dry matter content of approximately 15%) at − 20 °C until further use.

For disruption experiments, 25 mL thawed cell paste was transferred to a 50 mL falcon tube and water was added up to 50 mL to wash the cells. After collecting the cells using a centrifuge at 4 °C and 4700*g* for 5 min, the washing step was repeated once. An autolysis treatment was carried out by incubating washed yeast cells in water (7.5% DM) at 55 °C for 20 h at 220 rpm without pH adjustment. A hydrolytic treatment with Glucanex was done using an identical cell suspension, which was supplemented with 1:200 (w/w) Glucanex with 200 mM NaOAc pH 6 (hydrolytic treatment with Glucanex), followed by incubation at 37 °C for 24 h at 220 rpm. Identical 7.5% DM cell suspensions were also subjected to a mechanical treatment (cell disruption by high-pressure homogenization) using a microfluidizer (Microfluidizer™ SIMATIC HMI LM20) at 30.000 psi, for three consecutive cycles. The cell suspensions subjected to autolysis, hydrolytic treatment with Glucanex, homogenization with a microfluidizer and to no treatment were frozen at − 80 °C, and then freeze dried using an Alpha 2–4 LD plus freeze drier (Martin Christ, Osterode am Harz. Germany) set at − 60 °C and 0.01 mbar for a minimum of 24 h until the samples were dry. All these experiments were performed in triplicates. The whole process flow of this study is shown in Supplementary Figure S1.

### Analytical methods

#### Cell dry weight (CDW)

Fermentation broth samples (50 mL for shake flasks, 25 mL for bioreactors) were centrifuged at 4700*g* for 5 min at 4 °C and the supernatant was collected for further analyses (sugars, organic acids, ethanol, protein). Then, the yeast biomass was washed twice with cold distilled water, frozen at − 80 °C and then freeze dried using an Alpha 2–4 LDplus freeze drier (Martin Christ, Osterode am Harz, Germany) at − 60 °C and 0.01 mbar vacuum for a minimum of 24 h until samples were dry. The dried cells were weighed to determine CDW, and were also used for analysis of protein content and amino acids.

#### Monosaccharides, organic acids and ethanol

Monosaccharides (d-glucose, d-xylose), organic acids (lactic acid, acetic acid) and ethanol present in the fermentation broth were analyzed by high-performance liquid chromatography (HPLC) with refractive index detection. The samples were diluted ten times with distilled water and 200 µL of the diluted sample was vacuum filtered using 96-well filter plates (0.45 μm). Samples were separated on a Rezex ROA-organic acid H+ , 300 × 7.8 mm (Phenomenex, Torrance, CA, USA) analytical column fitted with a cation-H cartridge guard column, using a column temperature of 65 °C, 5 mM H_2_SO_4_ as eluent and a flow rate of 0.6 mL/min.

#### Protein content

The total nitrogen content of the poultry hydrolysates and the freeze-dried yeast biomass was measured according to the Kjeldahl method (European Commission [EC] regulation No: 152/2009, pp 15–19) using a Kjeltec TM 8400 (FOSS, Tecator, Hoganas, Sweden) after acid digestion in an autodigestor (FOSS, Tecator, Hoganas, Sweden). The protein content of samples was estimated by multiplying total nitrogen by a factor of 6.25.

#### Amino acids

Analysis of the content of amino acids (except tryptophan) in freeze-dried yeast was performed according to EC regulation No: 152/2009 (pp. 23–32) using a Biochrom 30 amino acid analyzer (Biochrom Ltd., Cambridge, UK). Tryptophan was analyzed according to EC regulation No: 152/2009 (pp. 32–37) using a Dionex Ultimate 3000 HPLC system (Dionex Softron GmbH, Germering, Germany) connected to a RF-535 fluorescence detector (Shimadzu., Kyoto, Japan). All amino acids were quantified using external standards (Dionex Ltd., Surrey, UK).

#### Scanning electron microscope (SEM)

For SEM images, the yeast cell suspensions were mixed with 37% (v/v) formaldehyde reaching a final concentration of 3.7%, and incubated at room temperature for 20 min. The yeast suspensions were then centrifuged for 5 min at 8000*g*, resuspended in 0.1 M potassium phosphate and kept at 4 °C until imaging. Prior to scanning electron microscopy, the cells were washed several times in 0.05 M Pipes buffer, pH 7.0, and dehydrated with 10 min stages in ascending ethanol series (30–100%). The samples were processed in a BAL-TEC Critical Point Dryer (CPD 030, Witten, Germany) and a thin conductive coating of gold/palladium was applied to the samples using a Polaron Sputter Coater (SC 7640, Kent, UK). The coated samples were mounted on brass stubs and examined and photographed with a Zeiss EVO-50-EP scanning electron microscope at an accelerating voltage of 15 kV in the secondary emission mode.

#### Statistical analysis

Data handling and statistics were performed using the Excel software package (Microsoft Excel 2013, Microsoft Corp., Redmond, WA).

## Results and discussion

### Characterization of BALI™ and poultry hydrolysates

Supplementary Table S1 shows that glucose is the main carbon source in the spruce BALI™ hydrolysates constituting 76% of the total sugar. The protein content of the protein-rich hydrolysates (named poultry hydrolysates in this study), based on the Kjeldahl method, was 444 ± 1 g/L. In the growth experiments described below, carbohydrates were dosed based on glucose, whereas the nitrogen source was dosed based on nitrogen.

### Preliminary 1.5 L batch fermentations using poultry hydrolysates

Initially, several batch fermentations were carried out at 1.5 L scale in bioreactors to compare the growth performance of the three yeast strains growing on a blend of BALI™ sugars and poultry protein hydrolysate. Supplementary Table S2 shows the CDW (g/L) and protein content (%) after 12 h and 24 h. In general, all the yeast strains had a protein content in the range 47–51% after 12 h of fermentation, but prolonged incubation somewhat reduced this. *C. jadinii* showed both the slowest growth rate and the lowest final production of microbial biomass. Both *W. anomalus* and *B. adeninivorans* grew faster and achieved a much higher biomass concentration after 24 h, in particular *B. adeninivorans* which reached a CDW of 44.0 g/L (as compared to 29.7 g/L and 18.0 g/L for *W. anomalus* and *C. jadinii*, respectively). This is in the same range as fed-batch cultures of *C. intermedia* growing on corncob hydrolysates which reached a CDW of 34.6 g/L after 40 h of incubation [26]. In another study, *C. jadinii* grown on rice polishing in batch reached a CDW of 50 g/L after 65 h [27]. A similar trend has been observed previously for these three yeast strains when grown on a medium composed of BALI™ sugars and an in-house prepared chicken hydrolysate (results not shown). Thus, in these batch fermentations, *B. adeninivorans* showed superior growth performance, probably due the good ability of this yeast to utilize peptides as a carbon source [28]. Of note, however, *B. adeninivorans* showed the lowest protein content after 24 h (41.9%).

### Assessing the ratio of organic and inorganic nitrogen

**Fig. 1:**
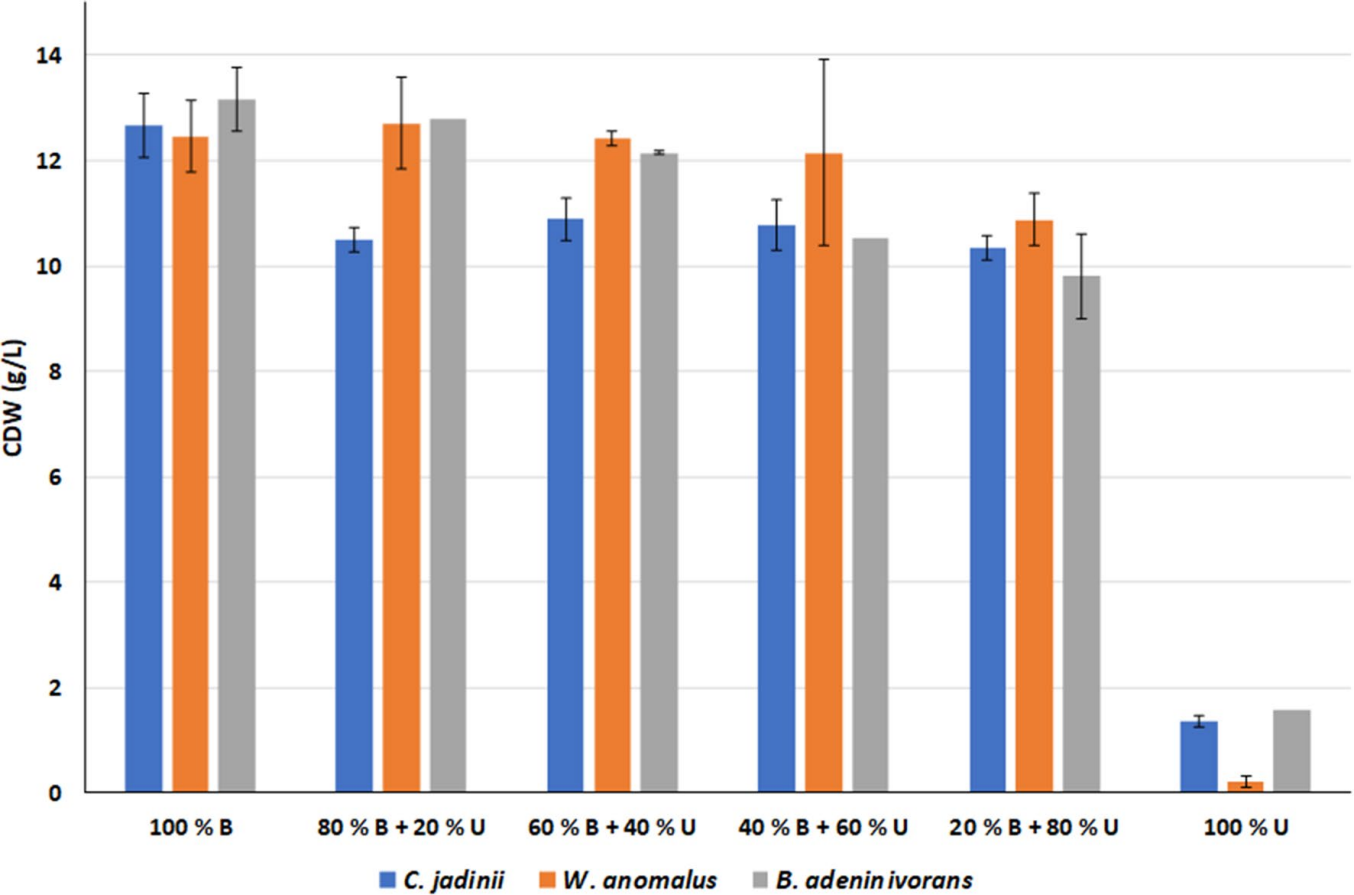
Growth of three yeast strains in shake flasks using six different media using different combinations of poultry hydrolysates and urea. The graph shows CDW (g/L) after 24 h cultivation (values are mean ± SD; *n* = 2). *B* BIOCO poultry hydrolysates, *U* Urea. Conditions: Glucose, 50 g/L; Kjeldahl nitrogen, 5.86 g/L; OD_initial_ = 0.5; volume: 50 mL; pH_initial_ = 5.0; incubation at 30 °C with 220 rpm shaking. The pH and pO_2_ were not controlled

**Fig. 2:**
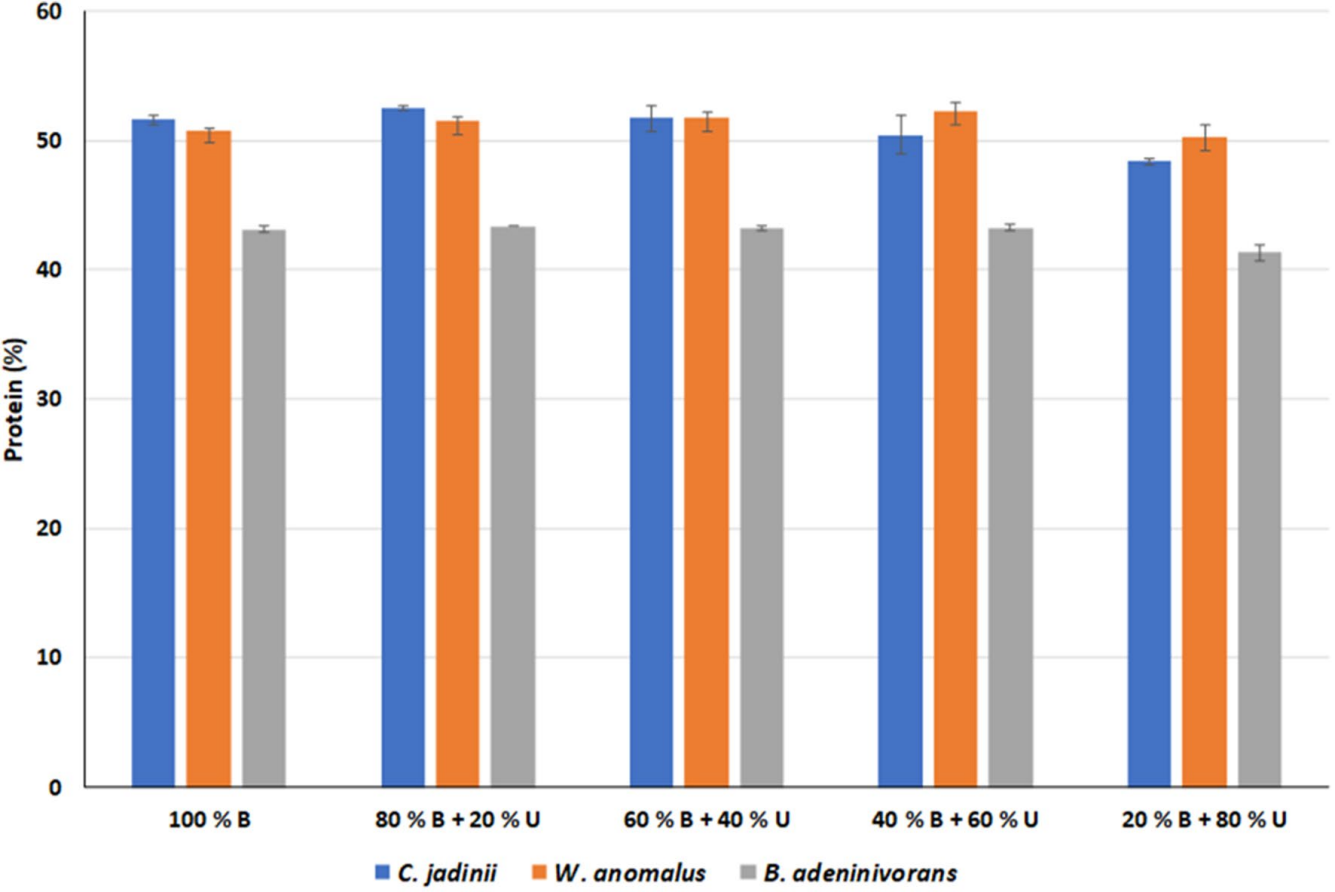
Protein content of three yeast strains grown in shake flasks using five different media using different combinations of poultry hydrolysates and urea. The graph shows protein content (%) after 24 h cultivation (values are mean ± SD; *n* = 2). *B* BIOCO poultry hydrolysates, *U* Urea. Conditions: Glucose, 50 g/L; Kjeldahl nitrogen, 5.86 g/L; OD_initial_ = 0.5; volume: 50 mL; pH_initial_= 5.0; incubation at 30 °C with 220 rpm shaking. The pH and pO_2_ were not controlled. The protein content in cells grown on urea only (see Fig. 1) was not determined

To test the importance of the protein hydrolysate for growth, a screening of growth was performed where different amounts of protein hydrolysate were substituted with inorganic nitrogen (urea). The experiments were conducted in shake flasks and the results in terms of CDW (g/L) and protein content (%) are shown in Figs. 1 and 2, respectively. After 24 h, the growth based on cell dry weight was between 9.8 and 13.2 g/L for all the yeasts and all fermentations containing poultry hydrolysates (100–20%). When only urea was used as a nitrogen source (condition 6), there was hardly any growth (note that the protein content for condition 6 could not be determined due to the lack of sufficient amounts of microbial biomass for the analysis). The observed growth yields were much lower than 50% (in this case, 25 g/L) which is typically achieved for yeast under aerobic conditions [29]. Analysis of the supernatants after 24 h showed that glucose was completely consumed for *C. jadinii* and *W. anomalus*, while *B. adeninivorans* did not consume all glucose (data not shown). Without protein hydrolysate in the medium, less than 20% of the glucose was consumed for any yeast. Ethanol concentrations between 17.0 and 24.7 g/L were observed for all the *C. jadinii* and *W. anomalus* cultivations containing poultry hydrolysates. For *B. adeninivorans*, ethanol concentrations were in the range of 4.8–11.7 g/L (data not shown). The pH was measured after 24 h (data not shown), and it was observed that the buffer capacity decreased when less poultry hydrolysate was included in the medium, with pH values after 24 h being 4.77, 4.36 and 4.50 in the cultures with 80% poultry hydrolysate and 20% urea, and 4.30, 3.89 and 3.32 in the cultures with 20% poultry hydrolysate and 80% urea, for *C. jadinii, W. anomalus*, and *B. adeninivorans*, respectively. In the cultures with only urea, the final pH values varied between 5.53 and 5.81. Acetic acid was also measured and concentrations ranged between 0 and 3 g/L (data not shown). All in all, these results show that the shake flask cultures were clearly limited regarding oxygen supply, causing high ethanol concentrations, and by medium acidification.

The general trends visible in Figs. 1 and 2 are that biomass production went down as the amount of poultry hydrolysates was reduced, whereas the protein content of the cells, around 50% for *C. jadinii* and *W. anomalus* and 43% for *B. adeninivorans,* was hardly affected. Although the differences in growth are likely primarily due to pH effects, the data in Figs. 1 and 2 do make clear that the cells can not simply convert urea to protein, but also that they need some of the components provided by the poultry hydrolysate (since on urea only growth was strongly restricted). It is well known that inorganic nitrogen can provide a considerable amount of nitrogen when combined with an organic nitrogen source that supplies additional benefits such as various trace elements [29, 30].

### 2.5L Bioreactor runs

Shake flask experiments suffer from a lack of pH control, as well as sub-optimal aeration (pO_2_) and stirring. Therefore, *C. jadinii*, *W. anomalus* and *B. adeninivorans* were cultivated in 2.5 L fermenters in repeated fed-batch mode. The main objectives were to identify the best medium ratios of poultry hydrolysate and urea to support high productivity (*Q*_*x*_), while producing yeast biomass containing around 50% protein [6, 18].

#### Using 100% poultry hydrolysates

We then carried out repeated fed-batch fermentations in 1.5 L fermenters with all three yeast, using 100% poultry hydrolysate as nitrogen source. Raw data from these fermentations are provided in Supplementary Figure S2, while Table 1 summarizes the key features. This procedure worked very well for *C. jadinii* and *W. anomalus* but not for *B. adeninivorans*, which left much of the glucose unused and, thus, showed low productivity. *C. jadinii* and *W. anomalus* used most of the glucose, showed good biomass productivity, amounting to 2.51 and 2.61 g/L/h, respectively. This is clearly higher than productivities of 1.15 and 0.86 g/L/h achieved for other candida strains produced on rice polishing [27] and corncob hydrolysates [26], respectively. For continuous cultures of *C. jadinii* using molasses as carbon source, a maximum productivity of 2.15 g/L/h has been reported [31]. The protein content of the cells produced in our study was 40.9% and 48.4% for *C. jadinii* and *W. anomalus,* respectively. While *C. jadinii* reached high cell densities, this came at the cost of lower protein content. A similar trade-off between cell mass and protein content was observed by Sharma et al. [32]. Overall*, W. anomalus* was clearly the best protein producer when grown in medium containing the poultry hydrolysate as the only nitrogen source.

**Table 1:** Growth characteristics for 1.5 L repeated fed-batch fermentations of *C. jadinii*, *W. anomalus* and *B. adeninivorans* grown on 100% poultry hydrolysates and BALI™ hydrolysates

	*C. jadinii*	*W. anomalus*	*B. adeninivorans*
Increase in CDW (g/L)^a^	20.05 ± 2.01	20.87 ± 2.44	14.69 ± 8.62
Protein (%)	40.89 ± 1.54	48.38 ± 0.91	47.44 ± 2.77
Increase in protein (g/L)^a^	8.18 ± 0.65	10.09 ± 1.09	6.99 ± 4.17
Unconsumed glucose (g/L)	2.90 ± 1.55	0.99 ± 0.77	25.06 ± 8.72
*Y*_*X*/glucose_^b^	0.48	0.54	0.50
*Y*_X/sugars_^c^	0.37	0.41	0.38
*Y*_p/glucose_^b^	0.20	0.26	0.24
*Y*_p/sugars_^c^	0.15	0.20	0.18
*Q*_*x*_^d^	2.51	2.61	1.51
*Q*_*p*_^e^	1.02	1.26	0.71

The *V*_out_/*V*_*f*_ was 0.75, every 8 h, and starting at 16 h. The data shown are average values for the samples taken at 24 h and at the end of the subsequent six repeated batches (32–72 h). The nitrogen concentration in the medium was 5.86 g/L, whereas the glucose concentration was approximately 50 g/L (the actual glucose concentrations were measured by HPLC at each sampling point and right after each harvest/refill procedure, and these measurements were used for the calculations). Supplementary Figure S2 provides the actual values of the parameters during the course of the fermentation

^a^Increase over the 8 h growth period following each harvest/refill procedure

^b^Yield of cell biomass (X) or protein (P) per consumed glucose

^c^Yield of cell biomass (X) or protein (P) per total added sugar

^d^Cell biomass productivity in g/L/h

^e^Protein productivity in g/L/h

#### Using 40% poultry hydrolysate and 60% urea

Inorganic nitrogen is cheaper than protein hydrolysates and may be easier to take up and metabolize. Thus, substituting part of the protein hydrolysate with inorganic nitrogen may be beneficial. The same set of fermentation experiments were repeated, but this time, 60% of the nitrogen in the protein hydrolysate was substituted with urea nitrogen. Compared to the fermentations using 100% poultry hydrolysates as N source, the cell biomass production substantially changed for *W.anomalus* (increased from 20.9 to 28.9 g/L) but the protein content decreased to 37.7% (Table 2; raw data in Supplementary Figure S3). For *C. jadinii*, the protein content increased to 44.9%, while biomass production was similar to the fermentation with 100% poultry. The *B. adeninivorans* fermentations showed better performance than in the fermentations with 100% poultry hydrolysate, but did still not consume glucose very well, indicating that the fermentation strategy still was not optimal for this yeast. In accordance with previous observations, *B. adeninivorans* performed better than expected on the basis of the low glucose consumption, which is likely due to the ability of this yeast to effectively use peptides in the poultry hydrolysate as carbon source [28].

**Table 2:** Growth characteristics for 1.5 L repeated fed-batch fermentations of *C. jadinii*, *W. anomalus* and *B. adeninivorans* grown on BALI™ hydrolysate with a mixture of 40% poultry hydrolysate and 60% urea as nitrogen source

	*C. jadinii*	*W. anomalus*	*B. adeninivorans*
Increase in CDW (g/L)^a^	21.08 ± 1.45	28.88 ± 1.11	18.51 ± 3.18
Protein (%)	44.87 ± 4.03	37.66 ± 2.19	44.45 ± 3.16
Increase in protein (g/L)^a^	9.43 ± 1.69	10.88 ± 0.89	8.19 ± 1.31
Unconsumed glucose (g/L)	3.48 ± 2.50	0.55 ± 0.13	25.53 ± 6.83
*Y*_X/glucose_^b^	0.55	0.69	0.89
*Y*_X/sugars_^c^	0.41	0.53	0.68
*Y*_p/glucose_^b^	0.24	0.26	0.39
*Y*_p/sugars_^c^	0.19	0.20	0.30
*Q*_*x*_^d^	2.63	3.61	2.31
*Q*_*p*_^e^	1.18	1.36	1.02

The *V*_out_/*V*_*f*_ was 0.75, every 8 h, and starting at 16 h. The data shown are average values for the samples taken at 24 h and at the end of the subsequent six repeated batches (32–72 h). The nitrogen concentration in the medium was 5.86 g/L, whereas the glucose concentration was approximately 50 g/L (the actual glucose concentrations were measured by HPLC at each sampling point and right after each harvest/refill procedure, and these measurements were used for the calculations). Supplementary Figure S3 provides the actual values of the parameters during the course of the fermentation

^a^Increase over the 8 h growth period following each harvest/refill procedure

^b^Yield of cell biomass (X) or protein (P) per consumed glucose

^c^Yield of cell biomass (X) or protein (P) per total added sugar

^d^Cell biomass productivity in g/L/h

^e^Protein productivity in g/L/h

All in all, comparing the results of Table 1 (100% poultry hydrolysate) and Table 2 (40% poultry hydrolysate) does not provide immediate clues for further optimization of the process. However, this comparison clearly shows that varying the ratio of the organic and the inorganic nitrogen source has considerable effects.

#### Using 60% poultry hydrolysates with 40% urea

The experiments described above showed that *W. anomalus* had the highest productivity regarding both biomass (*Q*_*x*_ in the range 2.61–3.61) and protein (*Q*_*P*_ in the range 1.26–1.36) production, although it must be noted that the higher productivities depicted in Table 2 come at the cost of a low protein content of the cells. We have previously seen that *B. adeninivorans* outperformed *C. jadinii and W. anomalus* during batch fermentations using BALI™ hydrolysates and in-house generated chicken hydrolysates, due to its ability to not only grow on sugars but also on different nitrogen sources [28, 33]. In the repeated fed-batch setup used in this study, *B. adeninivorans* had not been able to consume the glucose during the 8 h cycles. Thus, in a next series of experiments, this time using 60% poultry and 40% urea as nitrogen source, the cycle time for *B. adeninivorans* was increased to 12 h in an attempt to obtain better glucose utilization. The results, summarized in Table 3 (raw data in Supplementary Figure S4), show that the longer cycle time indeed resulted in increased consumption of glucose and that glucose consumption was more stable. However, sugar consumption was still not complete and, moreover, performance wise (yields, productivities, protein content; see Table 3), the longer cycle time did not improve the overall process. For *W anomalus*, the change from 40 to 60% poultry hydrolysate resulted in improved performance such as a higher protein content (41.2% versus 37.7%) and higher productivity of both cell biomass (3.72 versus 3.61 g/L/h) and protein (1.53 versus 1.36 g/L/h).

**Table 3:** Growth characteristics for 1.5 L repeated fed-batch fermentations of *W. anomalus* and *B. adeninivorans* grown on BALI™ hydrolysate with a mixture of 60% poultry hydrolysate and 40% urea as nitrogen source

	*W. anomalus*	*B. adeninivorans*
Increase in CDW (g/L)^a^	29.78 ± 3.51	27.62 ± 1.30
Protein (%)	41.22 ± 1.19	42.45 ± 1.12
Increase in protein (g/L)^a^	12.24 ± 1.14	11.73 ± 0.67
Unconsumed glucose (g/L)	0.15 ± 0.03	11.37 ± 2.83
*Y*_X/glucose_^b^	0.71	0.81
*Y*_X/sugars_^c^	0.54	0.61
*Y*_p/glucose_^b^	0.29	0.34
*Y*_p/sugars_^c^	0.22	0.26
*Q*_*x*_^d^	3.72	2.30
*Q*_*p*_^e^	1.53	0.98

The *V*_out_/*V*_*f*_ was 0.75, every 8 h (*W. anomalus*) or every 12 h (*B. adeninivorans*), and starting at 16 h. The data shown are average values for the samples taken at 24 h and 28 h, respectively, and at the end of the subsequent six (*W. anomalus*) or four (*B. adeninivorans*) repeated batches. The nitrogen concentration in the medium was 5.86 g/L, whereas the glucose concentration was approximately 50 g/L (the actual glucose concentrations were measured by HPLC at each sampling point and right after each harvest/refill procedure, and these measurements were used for the calculations). Supplementary Figure S4 provides the actual values of the parameters during the course of the fermentation

^a^Increase over the 8 h growth period following each harvest/refill procedure

^b^Yield of cell biomass (X) or protein (P) per consumed glucose

^c^Yield of cell biomass (X) or protein (P) per total added sugar

^d^Cell biomass productivity in g/L/h

^e^Protein productivity in g/L/h

#### Using 60% poultry hydrolysate and 40% urea with biotin, and 80% poultry hydrolysate with 20% urea

In the experiments described above, *W. anomalus* was superior in terms of productivity, but in several of the fermentations, the protein content of the cells was rather low. In particular, while *W. anomalus* grew well on urea, replacement of poultry hydrolysate with urea led to lowered protein contents (e.g. 48.4% with 100% poultry hydrolysate, versus 37.7% with 40% poultry hydrolysate and 60% urea). Yeasts can assimilate urea in two different ways, either via the action of an extracellular urease leading to ammonia production or via import of urea and subsequent assimilation through the urea and amydolyase pathway [34]. In this latter case, addition of biotin is necessary since it works as a cofactor of the urea amidolyase [34]. Therefore, we carried out an additional experiment with the medium composed of 60% poultry hydrolysate and 40% urea and added 0.4 mg of biotin per gram of urea. Additionally, a repeated fed-batch fermentation was run using a medium composed of 80% poultry hydrolysate and 20% urea, without addition of biotin. Supplementary Figure S5 and Table 4 show that the addition of biotin had no significant effect on the production of cell biomass and the protein content, which were 30.00 g/L and 40.6%, respectively, compared to 29.8 g/L and 41.2% for the similar experiment without added biotin (Table 3).

**Table 4:** Growth characteristics for 1.5 L repeated fed-batch fermentations of *W. anomalus* grown on BALI™ hydrolysate with two different mixtures of poultry hydrolysate and urea as nitrogen source (60–40, with added biotin or 80–20)

	*W. anomalus*	
	Poultry hydrolysate 60% + UREA 40% + Biotin	Poultry hydrolysate 80% + UREA 20%
Increase in CDW (g/L)^a^	30.00 ± 2.07	27.04 ± 1.55
Protein (%)	40.61 ± 0.48	45.03 ± 0.81
Increase in protein (g/L)^a^	12.18 ± 0.75	12.17 ± 0.53
Unconsumed glucose (g/L)	0.19 ± 0.01	0.19 ± 0.03
*Y*_X/glucose_^b^	0.65	0.62
*Y*_X/sugars_^c^	0.49	0.47
*Y*_p/glucose_^b^	0.26	0.28
*Y*_p/sugars_^c^	0.20	0.21
*Q*_*x*_^d^	3.75	3.38
*Q*_*p*_^e^	1.52	1.52

The *V*_out_/*V*_*f*_ was 0.75, every 8 h, starting at 16 h. The data shown are average values for the samples taken at 24 h and at the end of the subsequent six repeated batches (32–72 h). The nitrogen concentration in the medium was 5.86 g/L, whereas the glucose concentration was approximately 50 g/L (the actual glucose concentrations were measured by HPLC at each sampling point and right after each harvest/refill procedure, and these measurements were used for the calculations). Supplementary Figure S5 provides the actual values of the parameters during the course of the fermentation

^a^Increase over the 8 h growth period following each harvest/refill procedure

^b^Yield of cell biomass (X) or protein (P) per consumed glucose

^c^Yield of cell biomass (X) or protein (P) per total added sugar

^d^Cell biomass productivity in g/L/h

^e^Protein productivity in g/L/h

Supplementary Figure S5 and Table 4 also show that the protein content increased to 45.0% after increasing the amount of poultry hydrolysates from 60 to 80%, while the production of cell biomass stayed at as high 27.0 g/L. The latter value is slightly lower than the value obtained with 60% poultry, but still much higher than the value of 20.9 g/L obtained with 100% poultry. Apparently, having some urea in the medium is highly favorable for cell biomass production. All in all, the run with 80% poultry hydrolysate seemed close to optimal, yielding productivity values of 3.38 and 1.52 g/L/h for cell biomass and protein, respectively.

### 42 L Bioreactor run

**Fig. 3:**
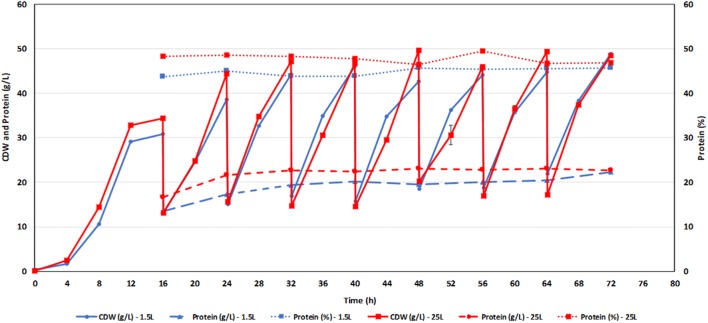
Data for the 1.5 L and 25 L repeated fed-batch fermentations of *W. anomalus* grown on a medium containing an 80:20 mixture of poultry hydrolysate and urea as nitrogen source and BALI™ hydrolysate as sugar source. The *V*_out_/*V*_*f*_ was 0.75, every 8 h, and starting at 16 h

Based on the observations and considerations described above, the combination of 80% poultry hydrolysate with 20% urea was selected as medium for upscaling the repeated fed-batch fermentation with *W. anomalus* from 1.5 to 25 L. Figure 3 shows that the 1.5 and 25 L fermentations behaved rather similar, but with somewhat higher *Q*_*x*_ (3.92 g/L/h) and *Q*_*P*_ (1.87 g/L/h), as well as a higher protein content (47.8%) in the large-scale fermentation (see Tables 4, 5). The biomass yield was 0.66 g biomass per g of glucose, which is in the higher range of yields report for aerobic growth of yeast, typically ranging from 0.4 and 0.5 g biomass per g of sugar [18].

**Table 5:** Growth characteristics for a 25 L repeated fed-batch fermentation of *W. anomalus* grown on BALI™ hydrolysate and an 80:20 mixture of poultry hydrolysate and urea

*W. anomalus*	
Poultry hydrolysate 80% + UREA 20%	
Increase in CDW (g/L)^a^	31.39 ± 2.77
Protein (%)	47.76 ± 1.13
Increase in protein (g/L)^a^	14.97 ± 1.07
Unconsumed glucose (g/L)	0.08 ± 0.01
*Y*_X/glucose_^b^	0.66
*Y*_X/sugars_^c^	0.50
*Y*_p/glucose_^b^	0.31
*Y*_p/sugars_^c^	0.24
*Q*_*x*_^d^	3.92
*Q*_*p*_^e^	1.87

The *V*_out_/*V*_*f*_ was 0.75, every 8 h, starting at 16 h. The data shown are average values for the samples taken at 24 h and at the end of the subsequent six repeated batches (32–72 h). The nitrogen concentration in the medium was 5.86 g/L, whereas the glucose concentration was approximately 50 g/L (the actual glucose concentrations were measured by HPLC at each sampling point and right after each harvest/refill procedure, and these measurements were used for the calculations). Figure 3 and Supplementary Figure S6 provide the actual values of the parameters during the course of the fermentation

^a^Increase over the 8 h growth period following each harvest/refill procedure

^b^Yield of cell biomass (X) or protein (P) per consumed glucose

^c^Yield of cell biomass (X) or protein (P) per total added sugar

^d^Cell biomass productivity in g/L/h

^e^Protein productivity in g/L/h

It is important to mention that it is difficult to compare biomass yields and productivity values for SCP production since they are strongly dependent on culture medium composition, the type of yeast and environmental conditions, such as incubation temperature, medium pH, dissolved oxygen, aeration rate and fermentation mode [4]. However, it is still possible to compare the efficiency of the 25 L experiment with *W. anomalus* with results obtained for well-known SCP yeasts such as *C. jadinii* (anamorph name *C. utilis*). Bajpai et al. [23] reached a *Q*_*x*_ 0.76 g/L/h, i.e., five times lower than in our study (3.92 g/L/h), for repeated fed-batch fermentations of *C. utilis* using the same ratio for the withdrawal and addition of medium (*V*_out_/*V*_*f*_ = 0.75) but with a longer cycle time of 24 h. Lee et al. [31] achieved biomass yields and productivities of 0.67 g/g and 0.24 g/L*h for batch fermentations, 0.51 g/g and 1.95 g/L*h for fed-batch fermentations, and 0.36 g/g and 2.15 g/L*h for continuous fermentations, using *C.utilis*. Gao et al. [35] produced single-cell protein (SCP) from soy molasses using *C. tropicalis* and obtained maximum cell densities and protein concentrations of 10.83 g/L and 6.11 g/L in a 10 L bioreactor, using batch fermentation. Overall, comparing the yields and productivity values from the literature with the values presented in Table 5, it can be concluded that it was possible to effectively produce a reasonable amount of protein-rich yeast in the form of *W. anomalus*, using a medium composed of spruce-derived components (BALI™ hydrolysates), poultry by-products and urea.

### Amino acid composition

The amino acid compositions of *W.anomalus* harvested during the 25 L fermentation, and of fish meal and soybean meal are presented in Table 6. Microorganisms to be used as feed ingredients would need a beneficial amino-acid profile, with particular attention to the nutritionally important amino acid methionine (Met), cysteine (Cys), lysine (Lys) and arginine (Arg) [36]. The data show that the amino acid composition of *W. anomalus* produced using 80% poultry hydrolysate and 20% urea with BALI™ sugar is similar to that of fishmeal and soybean meal, except for sulfur-containing amino acids such as methionine (Met) and cysteine (Cys). Low levels of sulfur-containing amino acids are common for yeast and bacterial biomasses [12] and normally restrict their use as the *sole* protein source in feed [37]. The data also show that the amino acid composition for *W. anomalus* is similar to the amino acid composition of the well-known feed ingredient *C. jadinii* grown on lignocellulosic substrates [27, 32, 38].

**Table 6:** Amino acid composition of *W. anomalus* obtained after repeated fed-batch fermentation on a medium containing an 80:20 mixture of poultry hydrolysate and urea as nitrogen source, and BALI™ sugar

Amino acids	*W. anomalus*	Fish meal^b^	Soybean meal^c^
EAAs^a^			
Met, M	3.27 ± 0.07	16.1	7.7
Thr, T	18.91 ± 0.05	25.4	20.2
Val, V	19.52 ± 0.13	26.4	24.1
Ile, I	18.41 ± 0.13	23.7	23.1
Leu, L	28.96 ± 0.05	42.0	39.0
His, H	11.19 ± 0.18	11.8	13.5
Lys, K	30.61 ± 0.23	45.5	32.3
Ala, A	24.11 ± 0.18	32.6	22.4
Phe, F	16.33 ± 0.06	22.0	26.5
Trp, W	5.20 ± 0.22	6.9	6.8
NEAAs^a^			
Asp, D	40.51 ± 0.15	54.7	59.5
Ser, S	20.19 ± 0.04	25.3	25.8
Glu, E	76.50 ± 0.48	83.9	92.1
Pro, P	17.67 ± 0.58	23.1	24.1
Gly, G	22.18 ± 0.08	30.8	21.6
Tyr, Y	11.20 ± 0.01	15.2	14.7
Arg, R	25.71 ± 0.04	35.3	37.4
Cys, C	3.27 ± 0.07	5.7	6.9
SUM AA	395.8	526.4	497.8

Values are mean ± SD (*n *= 2). *EAAs* essential amino acids, *NEAAs* non-essential amino acids

^a^ All values are in g/kg of dry matter

^b^The content of amino acids in fish meal (except tryptophan) was taken from Hansen et al. [46]; the value for tryptophan comes from Skrede et al. [47]

^c^The content of amino acids in soybean meal was taken from Sriperm et al. [48]

### Effects of different disruptive methods on *W. anomalus*

**Fig. 4:**
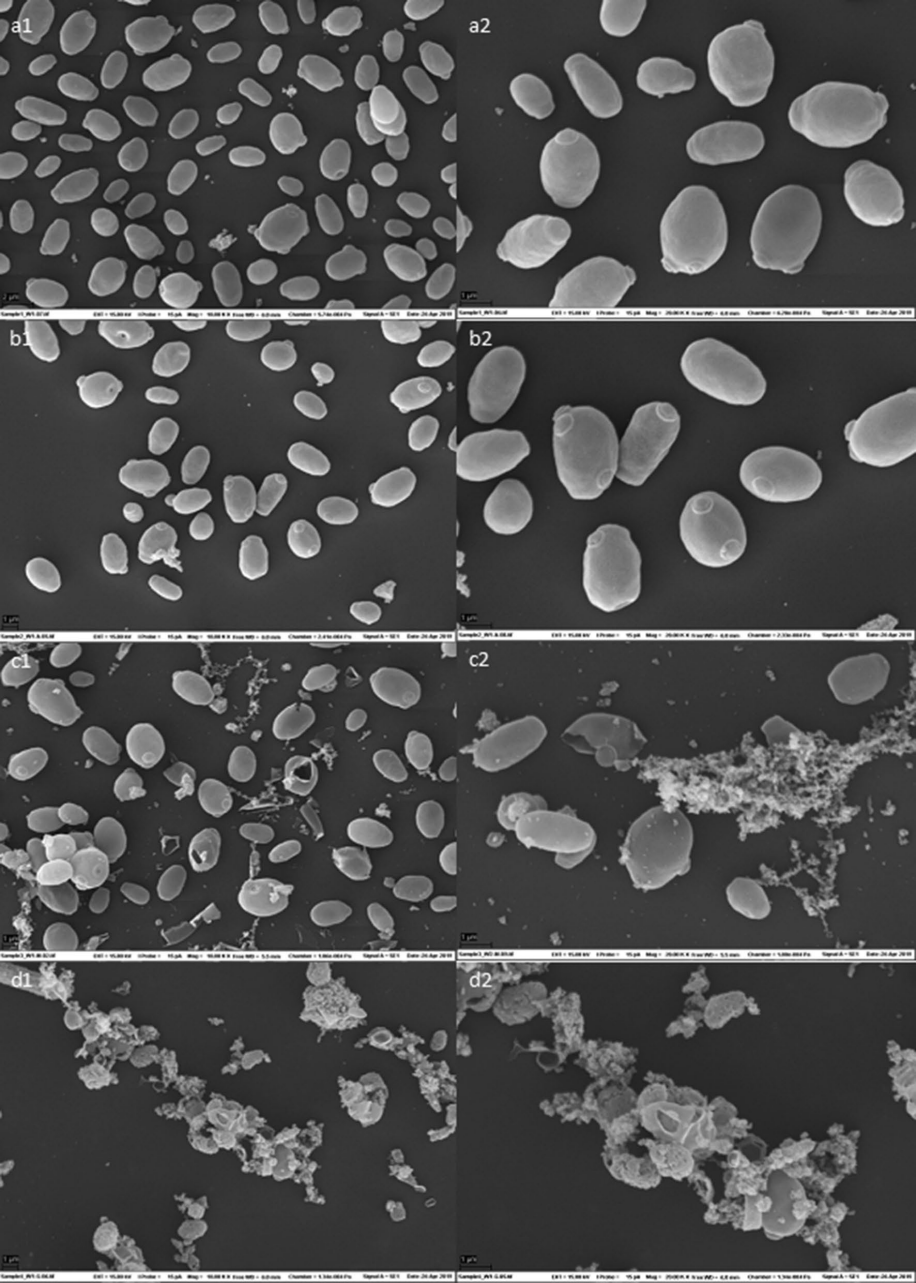
SEM images of *W. anomalus* cells after different potentially disruptive treatments. **a** Untreated yeast; **b** autolysis; **c** homogenization using a microfluidizer; **d** enzymatic hydrolysis using Glucanex. For each treatment, two magnifications are shown, 10,000 (panels labeled 1) and 20,000 (panels labeled 2)

*W. anomalus* has not been used in SCP production and little is known about how this yeast responds to downstream processing processes that are commonly in refining of SCP. To obtain some first insight into this issue, *W. anomalus* cells from the 25 L fermentation were subjected to varying potential processing steps. Cells from different harvesting points during the 25 L fermentation (16, 24, 32, 40, 48, 56, 64 and 72 h) were pooled and subjected to separation using a continuous two-phase separator, resulting in a yeast cell paste with a dry weight of 15% (w/v). After washing the cells, as described in the Materials and Methods section, they were subjected to varying disruptive methods followed by analysis of effects of on cell morphology using SEM. The SEM images of the autolyzed cells (Fig. 4b) did not differ much from the images of the untreated cells (Fig. 4a), while loss of cell integrity and liberation of internal contents were clearly visible in the pictures of homogenized cells (Fig. 4c). The SEM images also show a clear disruptive effect provoked by the use of the enzyme preparation Glucanex (Fig. 4d). These results indicate that several methods can be applied to disrupt the *W. anomalus* cells, which presumably would affect yeast digestibility. Such effects will be investigated in a follow-up study.

### Possible industrial applications as fish feed

The three major feed companies in Norway jointly used 1.63 million tons of ingredients for production of fish feed in 2012 [39]. These ingredients are mainly of plant and marine origin, which combined yield a feed protein content of approximately 50% (815,000 tons) [40]. If the SCP process developed in this study was to replace 10% of fish feed protein (81,500 tons), a total fermentation volume of approximately 5000 m^3^ would be needed, based on the results from the large-scale fermentation (protein productivity of 1.87 g/L/h or 0.016 tons/L/year). The commercial yeast producer Lallemand Inc. uses bioreactors with volumes of 100–300 m^3^ for production of baker’s yeast [41]. If bioreactors of 300 m^3^ (assuming a working volume of 200 m^3^) were used for production of SCP, a yearly production of 81,500 tons of fish protein would require 25 of such vessels (see Supplementary Table S3 for the numbers on which this and the subsequent calculations are based).

Regarding the raw materials, production of 81,500 tons of protein would annually require approximately 0.33 million tons of protein from poultry hydrolysate, 12,550 tons of urea and 220,000 tons of cellulose, which translates to 0.96 million tons of wet spruce [42, 43], which would amount to 11.3% of the total annual spruce harvest in Norway [44]. Of note, while the poultry hydrolysate:urea ratio was somewhat optimized in this study, we did not look into how to minimize the amount of the poultry hydrolysate–urea mixtures used. Thus, some further improvements in process economy should be feasible.

## Conclusions

In conclusion, this study demonstrates that *W. anomalus* is better suited than and *B. adeninivorans* and the well-established *C. jadinnii* to produce microbial protein in a medium composed of a mixture of organic and inorganic nitrogen sources and spruce-derived sugars in a semi-continues mode. The protein content of the yeast biomass produced in the 42 L fermenter was high (around 50 w/w%), and the amino-acid profile of *W. anomalus* was reasonable, albeit with the deficit in sulfur-containing amino acids, which is common for yeast and bacterial biomasses. The inclusion of *W. anomalus* has shown promising results in feeding experiments with rainbow trout [45]. However, further feeding experiments with *W. anomalus* as SCP in diets for animals and fish are needed to establish the full potential of the protein-rich *W. anomalus* cells produced by the protocols described in this study. It will also be important to investigate how different downstream processing routes, including cell disruptive methods, affect protein digestibility and nutritional value in animals and fish.

Additional research efforts may also be needed to further optimize and develop a more economically viable yeast production process based on industrial side-streams as substrates, in combination with cheap inorganic nitrogen sources. The poultry by-product hydrolysates used in this study are probably not best suited for SCP production, because they may find higher value applications in other markets, such as in food. Continuous fermentation modes may be worth further exploration, since these are also considered to be good strategies for microbial biomass production [18].

## Electronic supplementary material

Below is the link to the electronic supplementary material.

Supplementary material 1 (PDF 552 kb)

## Abbreviations

SCP: Single-cell protein
YP: Yeast extract and meat peptone
CDW: Cell dry weight
HPLC: High-performance liquid chromatography
ICS: Ion chromatography system
ICP-MS: Inductively coupled plasma mass spectrometry
AA: Amino acid
EAA: Essential amino acid
NEAA: Non-essential amino acid
*Y*_X/sugars_: Yield, g dry yeast per g sugar fed (g/g)
*Y*_P/sugars_: Yield, g yeast protein per g sugar fed (g/g)
*Y*_X/glucose_: Yield, g dry yeast per g consumed glucose (g/g)
*Y*_P/glucose_: Yield, g yeast protein per g consumed glucose (g/g)
*Q*_*x*_: Productivity, g dry yeast per liter and hour (g/L/h)
*Q*_*p*_: Productivity, g yeast protein per liter and hour (g/L/h)
