# Supplementary material for: Spruce sugars and poultry hydrolysate as growth medium in repeated fed-batch fermentation processes for production of yeast biomass
Source: Bioprocess Biosyst Eng. 2019 Dec 27;43(4):723–36. doi: 10.1007/s00449-019-02271-x (PMC7064453; doi:10.1007/s00449-019-02271-x)
Supplement: Supplementary file 1 — Supplementary material 1 (PDF 552 kb) [file 449_2019_2271_MOESM1_ESM.pdf]

Spruce sugars and poultry hydrolysate as growth medium in repeated fed-batch fermentation processes for production of yeast biomass

David Lapeña[1], Pernille M. Olsen[1], Magnus Ø. Arntzen[1], Gergely Kosa[1], Volkmar Passoth[2], Vincent G.H. Eijsink[1] and Svein J. Horn[1*],

[1] *Faculty of Chemistry, Biotechnology and Food Science, Norwegian University of Life Sciences (NMBU), P.O. Box 5003, N-1432 Ås, Norway*

[2] *Department of Molecular Sciences, Swedish University of Agricultural Sciences, P.O. Box 7015, S-75007 Uppsala, Sweden*

*Corresponding author; email: svein.horn@nmbu.no

Supplementary material

Supplementary material for *Spruce sugars and poultry hydrolysate as growth medium in repeated fed-batch fermentation processes for production of yeast biomass*

Contents:

Figure S1. Illustrated process flow of study.

Figure S2. Repeated fed-batch cultivation of three yeast strains grown on 100% poultry hydrolysates and BALI^{TM} hydrolysates in a 2.5 L fermenter

Figure S3. Repeated fed-batch cultivation of three yeast strains grown on 40% poultry hydrolysates and 60 % urea with BALI^{TM} hydrolysates in a 2.5 L fermenter

Figure S4. Repeated fed-batch cultivation of two yeast strains grown on 60% poultry hydrolysates with 40 % urea and BALI^{TM} hydrolysates in a 2.5 L fermenter

Figure S5. Repeated fed-batch cultivation of *W. anomalus* grown on 60% poultry hydrolysates, 40% urea and biotin or 80% poultry hydrolysates, 20% urea with BALI^{TM} hydrolysates in a 2.5 L fermenter

Figure S6. Repeated fed-batch cultivation of *W. anomalus* grown on 80% poultry hydrolysates, 20% urea with BALI^{TM} hydrolysates in a 42 L fermenter

Table S1 Composition of BALI^{TM} spruce hydrolysate

Table S2. CDW and protein content in 1.5 L batch fermentations (24h)

Table S3. The numbers and assumptions related to the industrial evaluation of replacing 10 % of the annual Norwegian fish feed protein by yeast produced using the media described in this study.

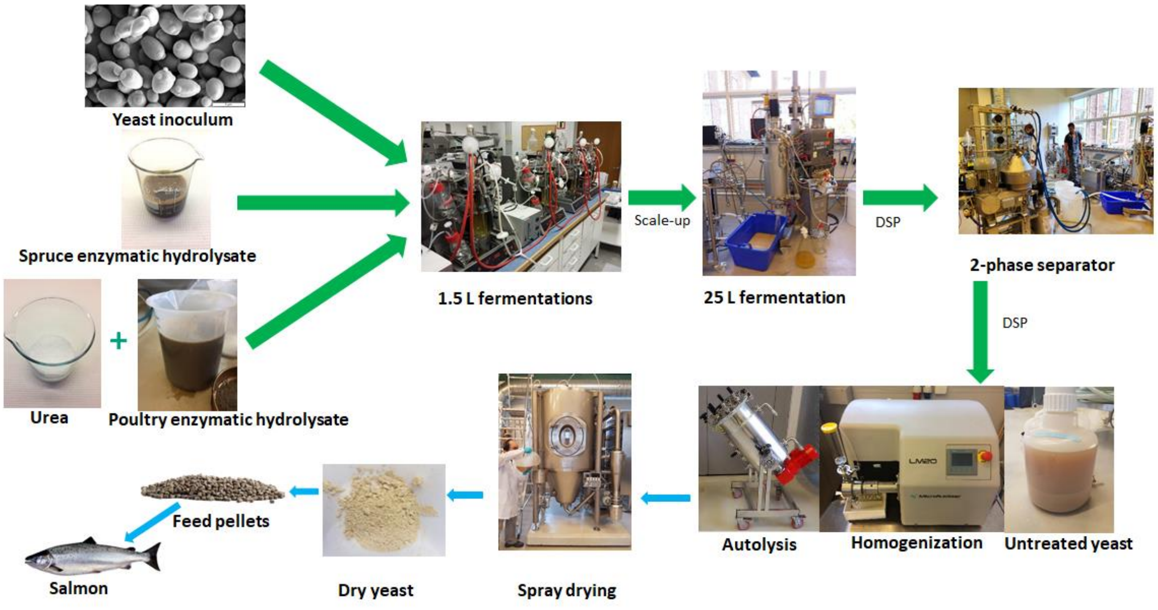

Figure S1. Process flow of study. Green arrows indicate processing steps included in this study. Blue arrows indicate planned further processing steps.

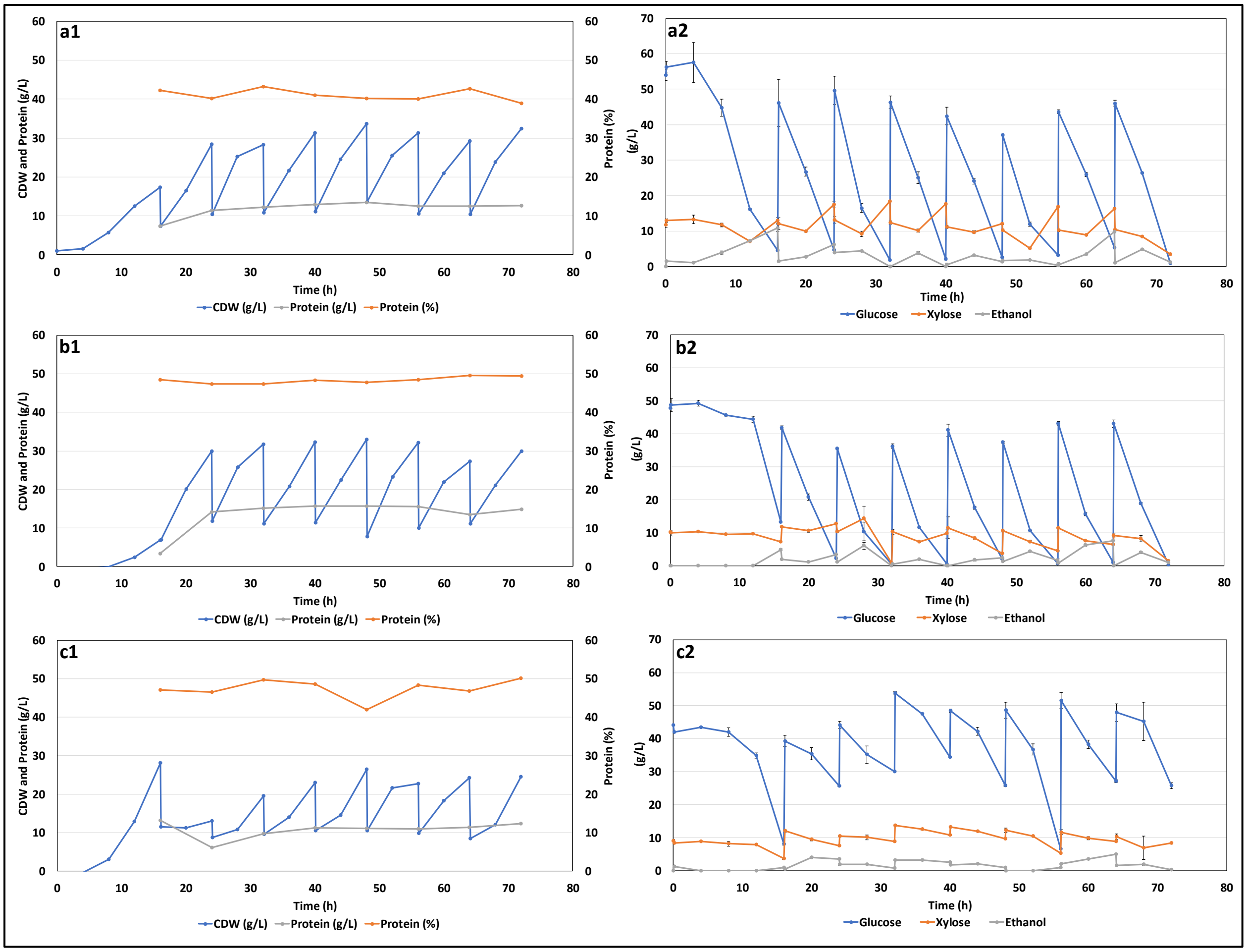

Figure S2. Repeated fed-batch cultivation of three yeast strains grown on 100% poultry hydrolysates and BALI™ hydrolysates in a 2.5 L fermenter. The starting volume was 1.5 L and the fermentation lasted 72 h. Panels labeled 1 show accumulation of cells (blue lines) and protein (grey lines), as well as the protein content of the cells (orange lines); panels labeled 2 show glucose (blue lines), xylose (orange lines) and ethanol (grey lines). a, *C. jadinii*; b, *W. anomalus*; c, *B. adeninivorans*. Growth was monitored by measuring the CDW (g/L, blue lines) every 4 h. For the samples taken from 16 h and onwards, the protein content (orange lines) of the dried cells was measured using the Kjeldahl method (w/w %). The concentration of yeast protein (g/L; grey lines) was calculated by multiplying CDW (g/L; blue lines) with the protein content (w/w %). Acetic acid and lactic acid production were negligible for all yeasts (results not shown).

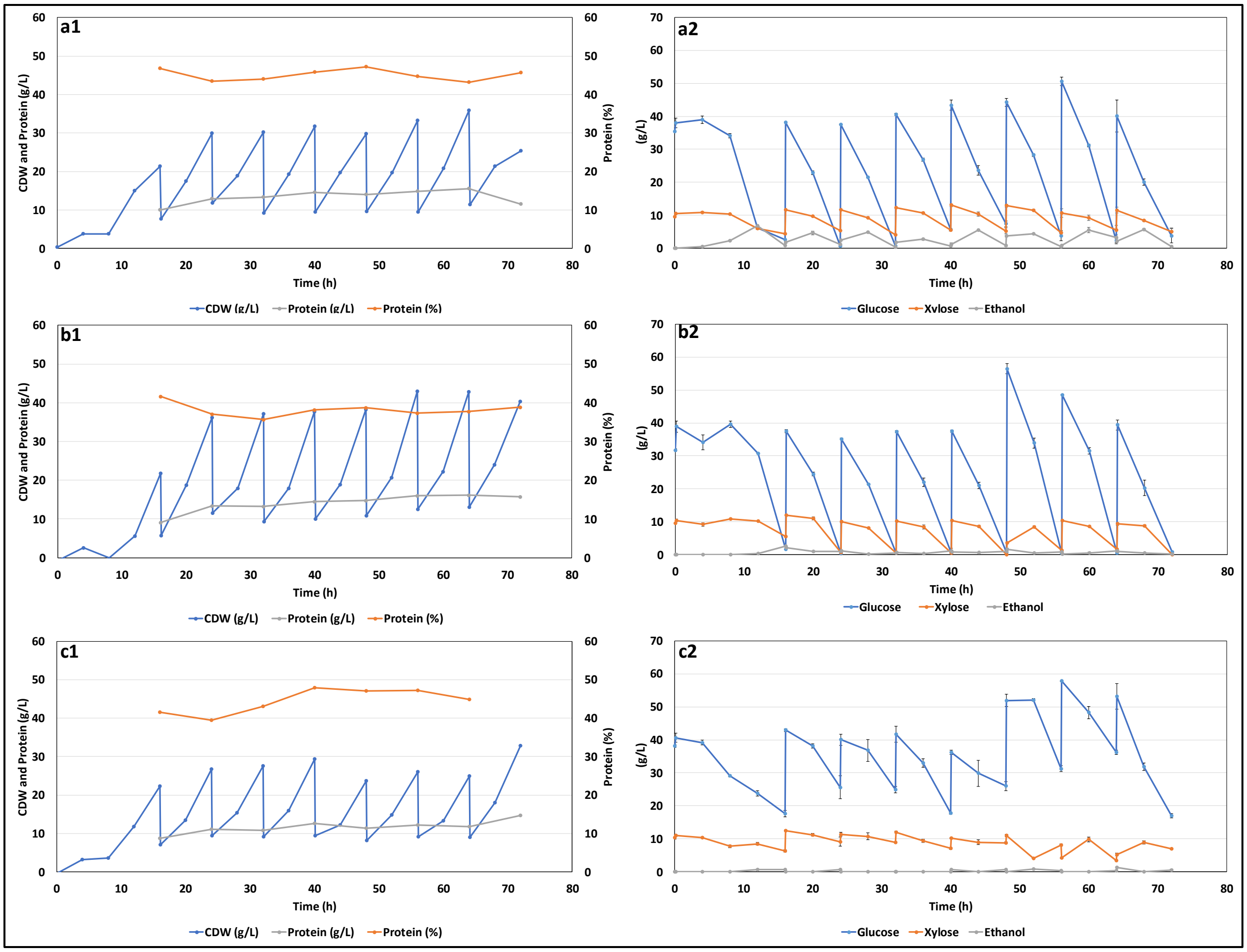

Figure S3. Repeated fed-batch cultivation of three yeast strains grown on 40% poultry hydrolysates and 60% urea with BALI^{TM} hydrolysates in a 2.5 L fermenter. The starting volume was 1.5 L and the fermentation lasted 72 h. Panels labeled 1 show accumulation of cells (blue lines) and protein (grey lines), as well as the protein content of the cells (orange lines); panels labeled 2 show glucose (blue lines), xylose (orange lines) and ethanol (grey lines). a, *C. jadinii*; b, *W. anomalus*; c, *B. adeninivorans*. Growth was monitored by measuring the CDW (g/L, blue lines) every 4 h. For the samples taken from 16 h and onwards, the protein content (orange lines) of the dried cells was measured using the Kjeldahl method (w/w %). The concentration of yeast protein (g/L; grey lines) was calculated by multiplying CDW (g/L; blue lines) with the protein content (w/w %). Acetic acid and lactic acid production were negligible for all yeasts (results not shown).

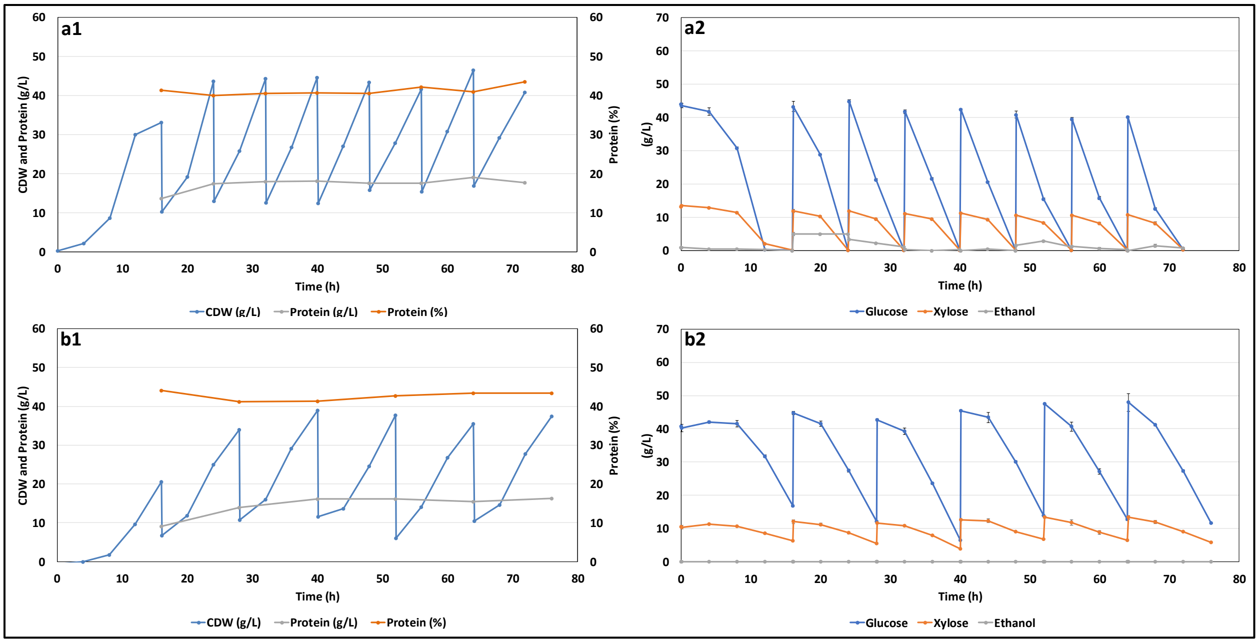

Figure S4. Repeated fed-batch cultivation of two yeast strains grown on 60% poultry hydrolysates with 40% urea and BALI^{TM} hydrolysates in a 2.5 L fermenter. The starting volume was 1.5 L and the fermentation lasted 72 h and 76 h, respectively. Panels labeled 1 show accumulation of cells (blue lines) and protein (grey lines), as well as the protein content of the cells (orange lines); panels labeled 2 show glucose (blue lines), xylose (orange lines) and ethanol (grey lines). a, *W. anomalus*; b, *B. adeninivorans*. Growth was monitored by measuring the CDW (g/L, blue lines) every 4 h. For the samples taken from 16 h and onwards, the protein content (orange lines) of the dried cells was measured using the Kjeldahl method (w/w %). The concentration of yeast protein (g/L; grey lines) was calculated by multiplying CDW (g/L; blue lines) with the protein content (w/w %). Acetic acid and lactic acid production were negligible for both yeasts (results not shown).

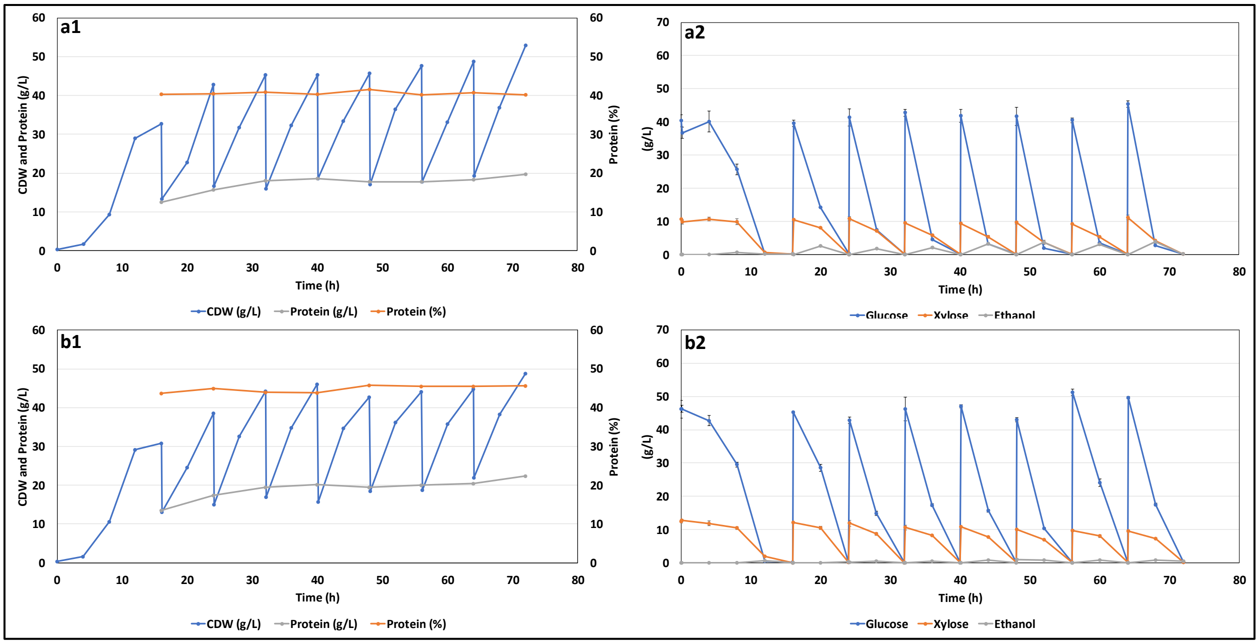

Figure S5. Repeated fed-batch cultivation of *W. anomalus* grown on 60% poultry hydrolysates, 40% urea and biotin or 80% poultry hydrolysates, 20% urea with BALI™ hydrolysates in a 2.5 L fermenter. The starting volume was 1.5 L and the fermentation lasted 72 h. a, 60% poultry hydrolysates, 40% urea and biotin b, 80% poultry hydrolysates, 20% urea. Panels labeled 1 show accumulation of cells (blue lines) and protein (grey lines), as well as the protein content of the cells (orange lines); panels labeled 2 show glucose (blue lines), xylose (orange lines) and ethanol (grey lines). Growth was monitored by measuring the CDW (g/L, blue lines) every 8 h. For the samples taken from 16 h and onwards, the protein content (orange lines) of the dried cells was measured using the Kjeldahl method (w/w %). The concentration of yeast protein (g/L; grey lines) was calculated by multiplying CDW (g/L; blue lines) with the protein content (w/w %). Acetic acid and lactic acid production were negligible on both media (results not shown).

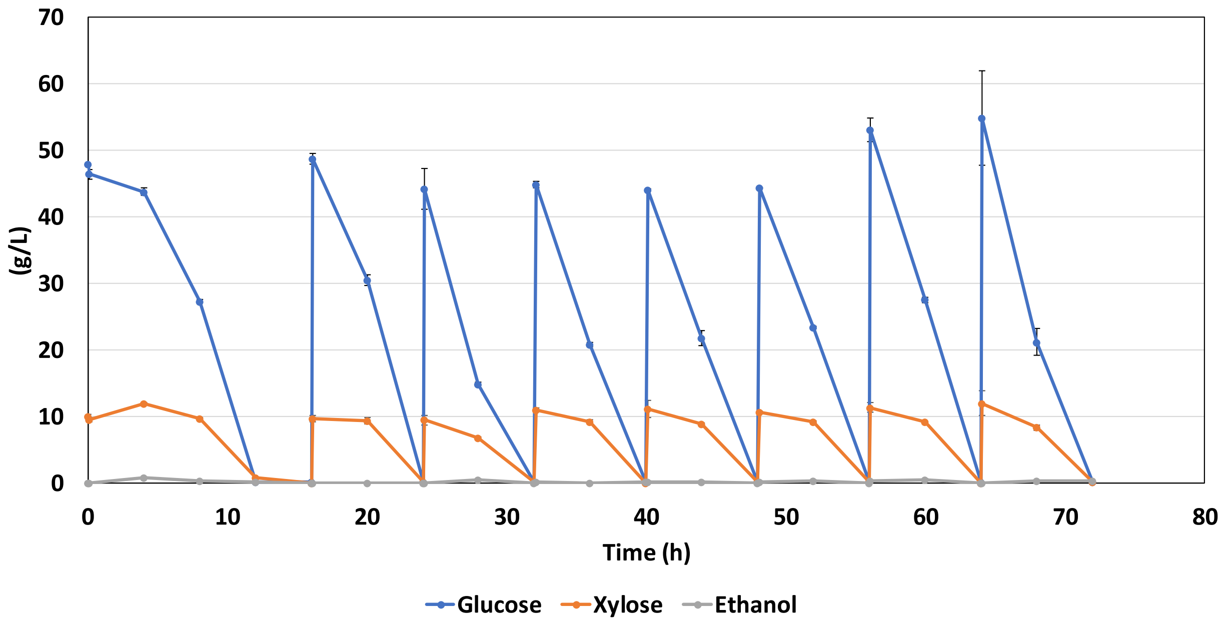

Figure S6. Repeated fed-batch cultivation of *W. anomalus* grown on 80% poultry hydrolysates, 20% urea with BALI™ hydrolysates in a 42 L fermenter. The starting volume was 25 L and the fermentation lasted 72 h. The figure shows glucose (blue lines), xylose (orange lines) and ethanol (grey lines). Acetic acid and lactic acid production were negligible (results not shown). Values are means ± SD (n = 2).

Table S1 Composition of BALI™ spruce hydrolysate

Content	BALI™
Dry matter (w/w %)	62.3
Density (kg/L)	1.286
Total sugars (% DM)	90.0
Glucose	68.2
Xylose	5.4
Mannose	6.5
Other sugars[a]	9.9
Acids[b]	1.2
Glycerol	0.2
Lignin	5.3

[a] Sum of fructose, arabinose, galactose, gentobiose and cellobiose. [b] Sum of lactic, formic and acetic acid.

Table S2. CDW and protein content in 1.5 L batch fermentations (24h).

		Poultry hydrolysates + BALI™	
	Sampling time (h)	CDW (g/L)	Protein content (%)
C. jadinii	12	13.4 ± 1.2	47.1 ± 0.7
	24	17.4 ± 1.7	45.1 ± 0.9
W. anomalus	12	17.7 ± 0.4	50.8 ± 0.4
	24	29.7 ± 0.2	45.9 ± 0.3
B. adeninivorans	12	16.4 ± 1.6	47.2 ± 0.4
	24	44.0 ± 0.0	41.9 ± 2.6

Table S3. The numbers and assumptions related to the industrial evaluation of replacing 10 % of the annual Norwegian fish feed protein by yeast produced using the media described in this study (a detailed description of the calculations is presented below).

	Quantity	Unit
Numbers related to replacing 10 % of fish feed by yeast: *		
Produced fish feed per year [1]	1 630 000	tonnes
Replace 10 % of the feed with SCP	163 000	tonnes
Biomass yield on glucose	0.66	g/g
Amount glucose needed for industrial production	250 000	tonnes
Amount of cellulose **	220 000	tonnes
Amount of dry spruce [2]	510 000	tonnes
Amount of wet spruce [3]	962 000	tonnes
% of the annual spruce harvest in Norway [4]	11.3	%
Amount BIOCO hydrolysate needed (80 %, 4.69 g N/L)	330 000	tonnes
Amount urea needed (20 %, 1.17 g N/L)	12 550	tonnes
Q_X*	3.92	g/L/h
Q_X (converted to tonnes per liter per year) *	0.03434	tonnes/L/year
Total fermentation volume required to produce 163 000 tonnes of SCP	4747	m^3
No. of bioreactors of 300 m^3 with a working volume of 200 m^3 required to produce the yeast	24	reactors
Numbers related to replacing 10 % of fish feed protein by yeast protein: *		
Amount feed protein per year (50% of the feed)	815 000	tonnes
Replace 10 % of the protein with SCP	81 500	tonnes
Q_P*	1.87	g/L/h
Q_P (converted to tonnes per liter per year) *	0.01638	tonnes/L/year
Total fermentation volume required to produce 81 500 tonnes of protein	4 975	m^3
No. of bioreactors of 300 m3 with a working volume of 200 m^3 required to produce the SCP	25	reactors

* Based data from the pilot-scale fermentation of *W. anomalus*. Note that the protein content of the yeast was 47.8 %, whereas the protein content of the feed is 50 %.

** Conversion of cellulose to glucose increases the mass by a factor 1.11. Note that the calculation assumes that saccharificaton of cellulose happens with 100 % efficiency.

Calculations for Table S3:

BALI Hydrolysate (Spruce)

$$Production\ of\ fish\ feed\ per\ year = 1\ 630\ 000\ tonnes$$

$$If\ 10\ \%\ is\ replaced$$

$$\frac{1\ 630\ 000\ tonnes}{10} = 163\ 000\ tonnes$$

$$The\ biomass\ yield\ based\ on\ glucose\ in\ our\ experiment\ is\ 0.66\ g/g$$

$$\frac{163\ 000\ tonnes\ SCP}{0.66\frac{g}{g}} \cong 250\ 000\ tonnes\ of\ glucose\ are\ needed$$

The Conversion from glucose to cellulose is 1.11 (180/162, Molecular weight ratio of free glucose to glucose units in cellulose)

$$\frac{250\ 000\ tonnes\ of\ glucose}{1.11} \cong 220\ 000\ tonnes\ of\ cellulose$$

$$The\ amount\ of\ cellulose\ in\ dried\ spruce\ is\ 43\ \%$$

$$\frac{220\ 000\ tonnes\ of\ cellulose}{0.43} \cong 510\ 000\ tonnes\ of\ dry\ spruce$$

$$The\ moisture\ content\ for\ Norwegian\ spruce\ is\ 53\ \%$$

$$510\ 000\ tonnes\ of\ dry\ spruce/0.53\ \cong 962\ 000\ tonnes\ of\ wet\ spruce$$

$$Around\ 10.0\ \times 10^6\ m^3\ (8.5\ \times 10^6\ wet\ tonnes)\ of\ spruce\ are\ annually\ harvested\ in\ Norway$$

$$Thus,$$

$$\frac{962\ 000\ tonnes\ of\ wet\ spruce}{8.5\ \times 10^6\ tonnes} \times 100\ \cong 11.3\ \%\ \text{of the annual spruce harvest in Norway}$$

BIOCO Hydrolysate:

$$Nitrogen\ originating\ from\ BIOCO\ in\ fermentations\ using\ 80\ \%\ BIOCO\ hydrolysat{:}\ \ 4.69\ g/L\ nitrogen$$

$$Nitrogen\ conversion\ factor\ (Kjeldahl\ method) =\ 6.25$$

$$4.69\ \left(\frac{g}{L}\ N\right) \times 6.25\ = 29.3\ \frac{g}{L}\ protein$$

$$Assuming\ 250\ \times 10^3\ tonnes\ of\ glucose\ are\ needed\ for\ industrial\ production$$

$$and$$

$$50\ \times\ 10^{-6}\ \frac{tonnes}{L}\ of\ glucose\ are\ used\ in\ our\ experiment{:}$$

$$The\ total\ fermentation\ volume\ \ would\ be$$

$$\frac{250\ \times 10^3\ tonnes\ of\ glucose}{50\ \times\ 10^{-6}\ \frac{tonnes}{L}} = 5000\ \times 10^6\ L$$

$$29.3\frac{g}{L}protein\ in\ fermentation\ from\ BIOCO \times\ 500\ \times 10^6\ L\ = 146\ 500\ tonnes\ of\ protein$$

$$= \mathbf{330\ 000}\ \boldsymbol{tonnes\ of BIOCO\ hydrolysate}\ (44\%\ protein)$$

Urea:

$$Nitrogen\ content\ in\ fermentations\ using\ 20\ \%\ Urea =\ 1.17\ g/L\ nitrogen$$

$$1.17\frac{g}{L}\ urea\ nitrogen\ was\ used\ for\ every\ 50\ \ \frac{g}{L}\ of\ glucose$$

$$60.06\ g\ urea\ in\ 1\ mol\ urea$$

$$28\ g\ nitrogen\ in\ 1\ mol\ urea$$

$$\frac{28\ g/mol}{60.06\ g/mol} = 0.466$$

$$1.17\frac{g}{L} nitrogen/0.466 = 2.51 \text{ g/L urea in the fermentations}$$

$$\frac{250\ \times 10^3\ tonnes\ of\ glucose\ x\ 2.51\ \frac{g}{L}\ urea}{50\ \frac{g}{L}\ glucose} \cong \mathbf{12\ 550\ tonnes\ of\ urea}$$

References

1) Ytrestøyl T, Aas TS, Åsgård T (2015) Utilisation of feed resources in production of Atlantic salmon (Salmo salar) in Norway. Aquaculture 448:365–374 . doi: 10.1016/j.aquaculture.2015.06.023

2) Bertaud F, Holmbom B (2004) Chemical composition of earlywood and latewood in Norway spruce heartwood , sapwood and transition zone wood. Wood Sci Technol 38:245–256 . doi: 10.1007/s00226-004-0241-9

3) Laurila J, Lauhanen R (2010) Moisture Content of Norway Spruce Stump Wood at Clear Cutting Areas and Roadside Storage Sites. Silva Fenn 44:427–434 . doi: 10.14214/sf.140

4) Landbruksdirektoratet (2109) Tømmeravvirkning og -priser. https://www.landbruksdirektoratet.no/no/statistikk/skogbruk/tommeravvirkning. Accessed 19 May 2019
